# AI-powered three-category *Helicobacter pylori* diagnosis via magnetic controlled capsule endoscopy: a multicenter validation of a vision-language model

**DOI:** 10.3389/fmicb.2025.1687021

**Published:** 2025-10-13

**Authors:** Xi Sun, Jing Liu, Lili Wu, Xiao Chen, Xiaona Ma, Fei Teng, Ting Zhang, Hui Su, Xin Fan, Jiaxin Li, Shiping Xu, Peng Jin, Hongmei Jiao

**Affiliations:** ^1^Department of Gastroenterology and Hepatology, The Second Medical Center of Chinese PLA Hospital and National Clinical Research Center for Geriatric Diseases, Beijing, China; ^2^The Institute of Geriatrics, The Second Medical Center of Chinese PLA Hospital and National Clinical Research Center for Geriatric Diseases, Beijing, China; ^3^Department of Gastroenterology, The Seventh Medical Center of Chinese PLA General Hospital, Beijing, China; ^4^Department of Geriatrics, Peking University First Hospital, Beijing, China

**Keywords:** *Helicobacter pylori*, artificial intelligence, gastric cancer, large language model (LLM), capsule endoscope

## Abstract

**Introduction:**

Accurate classification of *Helicobacter pylori* (*H. pylori*) infection status is critical for gastric cancer risk stratification. Current methods based on traditional convolutional neural networks (CNNs) are limited by their reliance on fragmented single-image analysis and operator-dependent selection variability, impairing diagnostic reliability.

**Methods:**

To overcome these limitations, we developed MC-CLIP, a vision-language foundation model for the fully automated, three-categorical diagnosis of *H. pylori* infection using magnetically controlled capsule endoscopy (MCCE). The model was first pretrained on a large-scale dataset of 2,427,475 MCCE image-text pairs derived from 123,543 examinations. It was subsequently fine-tuned on 40,695 expertly annotated images from 864 patients. MC-CLIP autonomously selects 30 representative images per case for end-to-end classification. Its performance was rigorously evaluated on multicenter internal (*n* = 220) and external (*n* = 208) validation cohorts.

**Results:**

On the internal and external validation cohorts, MC-CLIP achieved overall accuracies of 89.6% (95% CI: 85.5–93.6%) and 86.6% (80.8–90.3%), respectively. The model demonstrated particularly high sensitivity in detecting *H. pylori infection*: 91.4% for current infection and 83.7% for past infection. This performance significantly surpassed that of both senior endoscopists (84.3% and 71.4%, respectively) and junior endoscopists (74.3% for current infection). MC-CLIP also maintained high specificity (>90% across all categories) and excelled at identifying subtle mucosal changes following eradication therapy, thereby reducing the misclassification of past infections as non-infections.

**Discussion:**

By integrating multimodal image-text data and performing end-to-end analysis, MC-CLIP effectively addresses the fundamental limitations of CNN-based approaches. The model shows strong potential for enhancing the accuracy and reliability of MCCE-based gastric cancer screening programs.

## Introduction

Gastric cancer (GC) now ranks as the 3^rd^ leading cause of cancer-related mortality worldwide, causing severe global health burdens, particularly in East Asian countries (Bray et al., 2018). Early detection of GC via esophagogastroduodenoscopy (EGD) and successful eradication of *H. pylori*, the primary carcinogen for GC, could effectively reduce GC-related mortality (Correa and Houghton, 2007).

Currently, technical advances allow endoscopic GC screening to be performed in a noninvasive and comfortable manner (Liao et al., 2016; Zou et al., 2015; Qian et al., 2018) A previous large population-based cross-sectional study demonstrated that magnetic controlled capsule endoscopy (MCCE) performed well in GC screening (Zhao et al., 2018).

Despite the safety and comfort of MCCE, patients who are diagnosed with GC or have findings that are highly suspicious for GC or high-grade precancerous lesions via MCCE still require a subsequent EGD to confirm the diagnosis. Thus, for MCCE population-based GC screening, risk stratification by accurately classifying *H. pylori* infection status into three categories, namely, current-infection, non-infection and past- infection (eradicated), is necessary. Our previous work showed that, on the basis of the Kyoto classification of gastritis (KCG), three-categorical diagnosis of *H. pylori* infection status could be performed well using MCCE, with an accuracy of 80.3%(Xi et al., 2022), comparable with that of an EGD study (Yoshii et al., 2020).

However, performing three-categorical diagnosis of *H. pylori* infection via MCCE is a challenging task, it not only is time-consuming but also requires a considerable level of expertise. The incorporation of artificial intelligence (AI) in clinical settings is anticipated to ameliorate this situation (Kaul et al., 2020). Convolutional neural network (CNN) model-based AI systems have been applied to various aspects of gastrointestinal endoscopy, including the diagnosis of *H. pylori* infection (Kaul et al., 2020; Dilaghi et al., 2022,; Jiang et al., 2025).

Despite their widespread use and excellent results for binary *H. pylori* infection diagnosis, CNN architectures such as ResNet-50 and Inception-v3 present inherent constraints for three-categorical *H. pylori* infection diagnosis (Shichijo et al., 2017; Nakashima et al., 2020; Seo et al., 2023; Li et al., 2025; Shichijo et al., 2019). Their single-image processing paradigm disregards contextual relationships between gastric regions, a critical flaw given that three-categorical diagnosis requires synthesizing findings from multiple anatomical sites (Shichijo et al., 2017; Nakashima et al., 2020; Seo et al., 2023; Li et al., 2025; Shichijo et al., 2019). Furthermore, the need for manual image selection creates operator-dependent variability, particularly for past eradication cases where subtle mucosal changes may be overlooked (Shichijo et al., 2017; Seo et al., 2023; Shichijo et al., 2019). These shortcomings highlight the necessity of end-to-end systems capable of analyzing comprehensive image sets while minimizing human intervention.

Rapid technical advancements in AI algorithms led to the advent of the large language model (LLM), a highly successful model worldwide (Chen et al., 2024a, 2024b; Dosovitskiy et al., 2010). An LLM, such as contrastive language–image pretraining (CLIP), is capable of simultaneously processing multiple languages and images, enabling end-to-end diagnosis (Chen et al., 2024a, 2024b; Dosovitskiy et al., 2010). Previous studies have shown that LLMs work well for computational pathology tasks (Chen et al., 2024a, 2024b), but whether they are suitable for determining a three-categorical *H. pylori* infection diagnosis remains unclear.

The aim of this study was to develop and validate a vision-language foundation model (MC-CLIP) for the automated three-categorical diagnosis of *H. pylori* infection status using MCCE. We envision the primary application of this model as an assistive tool for endoscopists. This output is designed to directly inform clinical decisions, such as initiating eradication therapy for “current-infection” or determining appropriate endoscopic surveillance intervals for “past-infection,” thereby integrating into the comfortable MCCE gastric cancer screening pathway to improve the efficiency.

## Methods

### Study design

This was a multicenter study approved by the ethics board of the Chinese PLA’s General Hospital (IRB No. 2021–674-02). Study participants were selected between December 2021 and May 2024. For the training cohort and internal validation cohort, we recruited individuals who presented to the second medical center of the PLA’s General Hospital for MCCE examination. The external validation cohort was recruited from two other tertiary centers (the seventh medical center of the PLA’s general hospital, Peking University First Hospital).

All recruited participants had undergone MCCE and either a urea breath test (UBT) or a serological test to screen for *H. pylori* antibodies before the study. The training cohort was retrospectively recruited from patients who underwent an MCCE examination between December 2021 and October 2023. For validation, both the internal and external validation cohorts were established by consecutively enrolling naturally distributed cases from January 2024 to August 2024.

Those who had gastric surgery, poor image quality, inadequate gastric preparation and those who had recently taken medications that affect gastric mucosa (such as antibiotics or proton pump inhibitor/PPI) were excluded from the study.

### MCCE procedure and definition of *H. pylori* infection status

The NaviCam MCCE system (Ankon Technologies (Wuhan) Co., Ltd.) was used for GC screening in this study, and all MCCE procedures were conducted according to the guidebook described in previous publications (Liao et al., 2016; Zou et al., 2015; Qian et al., 2018).

The definition of the three categories of *H. pylori* infection status was as follows: individuals who had a UBT lower than 4.0 U/mL and who claimed no *H. pylori* eradication history and who tested negative for the serological antibody were defined as “non-infection” whereas those who claimed an eradication history and tested positive for the serological antibody were defined as “past-infection.” Individuals with a UBT equal to or greater than 4.0 U/mL were classified as “current-infection,” regardless of their eradication history.

### Training dataset

**Figure 1:**
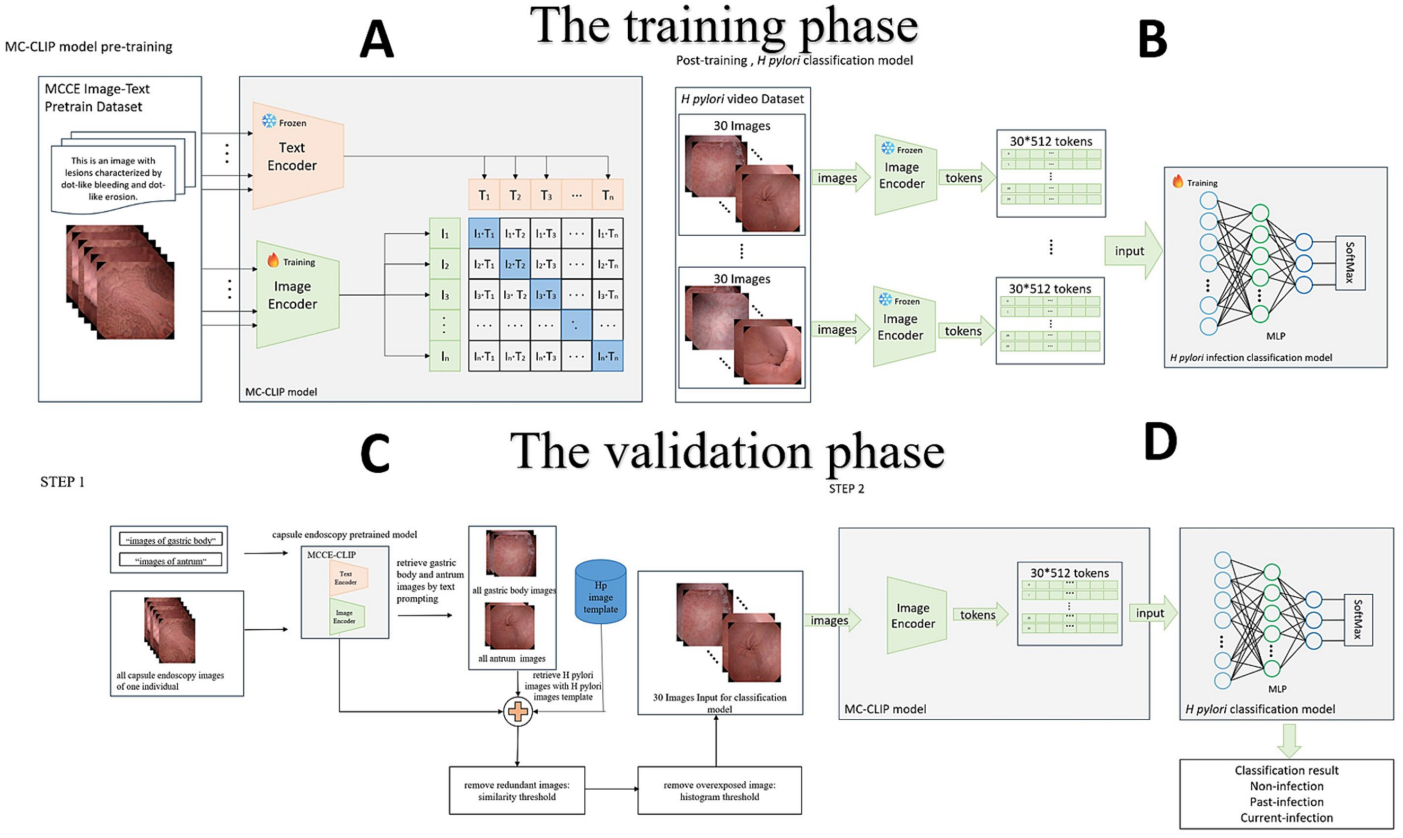
**(A)** Construction of the CLIP, which includes an image encoder and a text encoder. **(B)** Fine-tuning of the CLIP, the building of the multilayer perceptron (MLP) model. **(C)** Step 1: Image selection. MC-CLIP automatically selects 30 images from the original MCCE data according to the *H. pylori* template. **(D)** Step 2: End-to-end diagnosis. The MLP outputs the per-patient result of the *H. pylori* infection three-categorical classification.

For the pretraining of the model, we extensively collected more than 2,427,475 MCCE image–text data from 123,543 MCCE cases (Table 1) to construct a contrastive language-image pretraining (CLIP) model. The CLIP model is a multimodal pretraining neural network developed by Open AI (Yan et al., 2023) dedicated to investigating the alignment relationship between images and text through comparative studies (Figure 1A). This MC-CLIP includes an image encoder and a text encoder (Figure 1) and was fine-tuned with the aim of maximizing the cosine similarity for correct text–image pairs and minimizing the cosine similarity for incorrect text–image pairs. We utilized three Nvidia A100 GPUs for model training, iterating over 50 epochs, and ultimately selected the optimal model as the pretrained model for MCCE images.

**Table 1:** Baseline characteristics and data distribution.

Characteristics	Pre-training	Post-training	Internal validation	External validation	*p*-value
Cases (*n*)	123,543	864	220	208	–
Image-text pairs	2,427,475				–
Total images		40,695	6,600	6,240	–
Images per case		47.1 ± 12.3	30 (fixed)	30 (fixed)	–
Age (years)		53.4 ± 12.2	53.6 ± 11.2	54.8 ± 11.3	0.327(n.s)
Male proportion		54.6%	52.5%	51.7%	0.742(n.s)
*H. pylori* status					
Non-infection (n,%)		384, 44.4%	101, 45.9%	94, 45.2%	0.832(n.s)
Past-infection (*n*, %)		224, 25.9%	49, 22.3%	51, 24.5%	
Current-infection (*n*, %)		256, 29.6%	70, 31.8%	63, 30.3%	
Data source	Archive of Ankon	Retrospective cohort	Retrospective cohort	Independent hospitals	

n.s, not significant.

The *H. pylori* classification model was subsequently established for post training. Two experts on MCCE extracted a total of 40,695 images of three categorical *H. pylori* infection status from the data of 864 individuals in the training cohort (Figure 1B). Disparities of annotation between two experts were solved through mutual discussion. According to the KCG (Xi et al., 2022; Yoshii et al., 2020), characteristic findings of *H. pylori* infection status are mostly located in the gastric body (diffuse redness, mucosal swelling, regular arrangement of collecting venules, RAC) and antrum (nodularity, map like redness). Images of these two locations were extracted to train the model.

Prior to training the *H. pylori* classification model, specifically the multilayer perceptron (MLP) model, we applied data augmentation techniques to every case in the *H. pylori* classification dataset. Each image was subjected to approximately 10-fold data augmentation using techniques such as random rotation, horizontal and vertical flipping, and random distortion. The images were encoded using the image encoder of the MC-CLIP model, resulting in 30*512 feature vectors that served as the input data for the *H. pylori* classification model (Supplementary materials).

### Validation dataset

Step one of dataset validation is image selection. On the basis of the MC-CLIP model described above, we used text prompts to retrieve all gastric body and antrum images from each individual’s MCCE data. These images were compared to a predefined *H. pylori* atlas (derived from the training set) for similarity retrieval, retaining those with a cosine similarity >0.96—a threshold selected based on pilot analyses achieving >95% concordance with expert-annotated images. After removing redundant or poorly exposed images, we selected 30 images, including 20 gastric body and 10 gastric antrum images (reflecting the Kyoto classification’s emphasis on these regions). If the initial pool exceeded 30 images, the top 30 by similarity were chosen; if fewer were available, the highest-similarity images were reused to ensure consistent input dimensions (Figure 1C).

Step two is image classification. We used an MLP, as previously described, to set up a classifier in which the MCCE images were categorized as non-infection, past-infection and current-infection. The feature vector dimension generated by the image encoder of MC-CLIP was 1*512. Feature extraction was performed on the 30 images selected in step one, creating a 30*512-dimensional feature vector. After simultaneously processing the 30 images, the MLP output the per-patient result of three categorical *H. pylori* infection classification (Figure 1D) (Other details are documented in Supplementary materials).

### Diagnostic performance of the senior and junior physicians

MCCE data from the internal validation cohort were sent to a senior physician (EGD experience>10.000 and MCCE experience >2000) and a junior physician (EGD experience 3,000 ~ 5,000 and MCCE experience <500) for three-categorical diagnosis of *H. pylori* infection status. Neither of the physicians had taken part in the training phase, and they were blinded to both the clinical results and the AI diagnosis. Each physician independently made their three-categorical diagnosis after reviewing the video and images of each MCCE case.

### Sample size calculation and statistical analysis

The sample size calculation was done using the R package (version 4.3.2). Based on our pilot data (Xi et al., 2022), the expected prevalence of *H. pylori* infection states was 45% non-infection, 30% current-infection, and 25% past-infection. To detect a minimum AUC difference of 0.10 between categories with 90% power (*α* = 0.05), we required 196 total validation cases.

The statistical analysis compared diagnostic performance metrics (sensitivity, specificity, PPV, and NPV) across different groups, with the results reported as percentages and 95% confidence intervals (CIs). Comparisons of these metrics between the MC-CLIP model and each endoscopist were performed using McNemar’s test for paired proportions, given that both assessments were made on the same set of patients. In addition to *p*-values, the risk difference (RD) with its 95% confidence interval (CI) was calculated to quantify the magnitude of the difference in performance metrics between the MC-CLIP model and the endoscopists. The RD was derived from the paired 2×2 contingency tables, and its CI was calculated using the Wald method.

For baseline characteristics, continuous variables (e.g., age) were compared using one-way analysis of variance (ANOVA), and categorical variables (e.g., sex distribution, *H. pylori* status) were compared using the Chi-square test. All statistical tests were two-tailed, and a *p*-value of less than 0.05 was considered statistically significant.

All statistical analyses, including McNemar’s test and the calculation of risk differences (RDs) with confidence intervals, were performed using R software (version 4.3.2; R Foundation for Statistical Computing). The analysis of paired proportions utilized the stats package (version 4.3.2; for McNemar’s test) and the PropCIs package (version 0.3–0; for calculating risk differences and confidence intervals from paired data). The confusion matrices were generated using Python (version 3.10.12) with the scikit-learn library (version 1.2.2).

## Results

### Recruitment and baseline characteristics of the study participants

For the training cohort, a total of 1,012 individuals who underwent MCCE examination between December 2021 and October 2023 were initially assessed for eligibility. After applying the exclusion criteria—which included a history of gastrectomy (*n =* 48), recent use of proton-pump inhibitors or antibiotics within 4 weeks prior to the examination (*n =* 67), and poor image quality insufficient for analysis (*n =* 33)—a final total of 864 eligible participants were included in the training cohort. Among them, 384 (44.4%) were non-infection, 224 (25.9%) were past-infection and 256 (29.6%) were current-infection.

**Figure 2:**
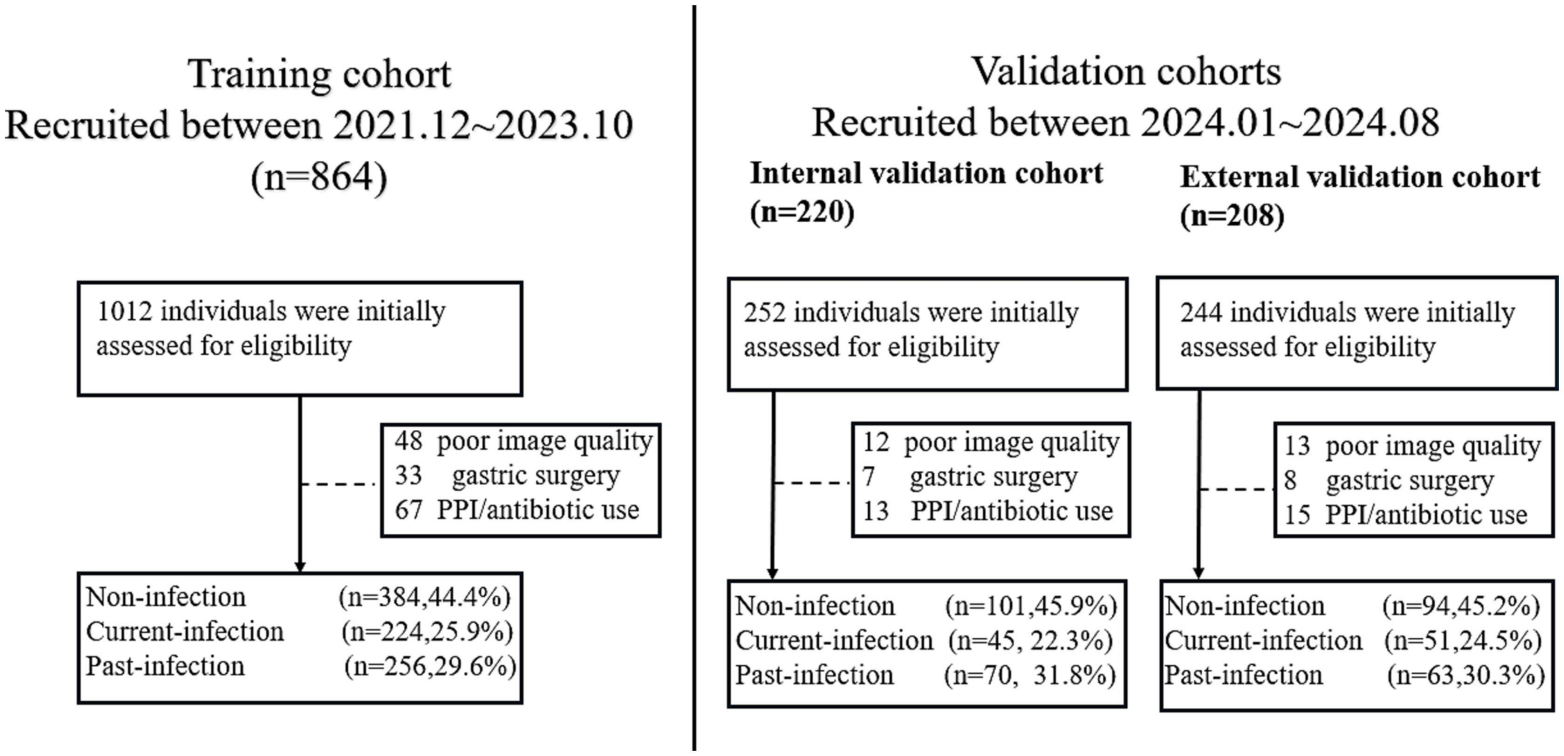
Flow diagram showing recruitment of study participants.

For the internal validation cohort, 252 consecutively enrolled cases from January 2024 to August 2024 were screened. Among them, 30 cases were excluded due to recent PPI/antibiotic use (*n =* 13), inadequate image quality (*n =* 12), or prior gastric surgery (*n =* 7), resulting in 220 eligible individuals. Among them, 101 were non-infection, 49 were past-infection, and 70 were current-infection. For the external validation cohort, 244 cases from the two participating tertiary centers were screened. Exclusions were made for recent medication use (*n =* 15), poor image quality (*n =* 13), and gastrectomy history (*n =* 8), yielding 208 eligible participants for the final external validation cohortcategorized into 94 non-infection cases, 51 past-infection cases, and 63 current-infection cases (Figure 2).

The average ages of the patients in the training cohort, internal validation cohort and external validation cohort were 53.2, 54.6 and 53.8 years, respectively (Table 1).

### MC-CLIP’s internal vs. external validation performance

**Figure 3:**
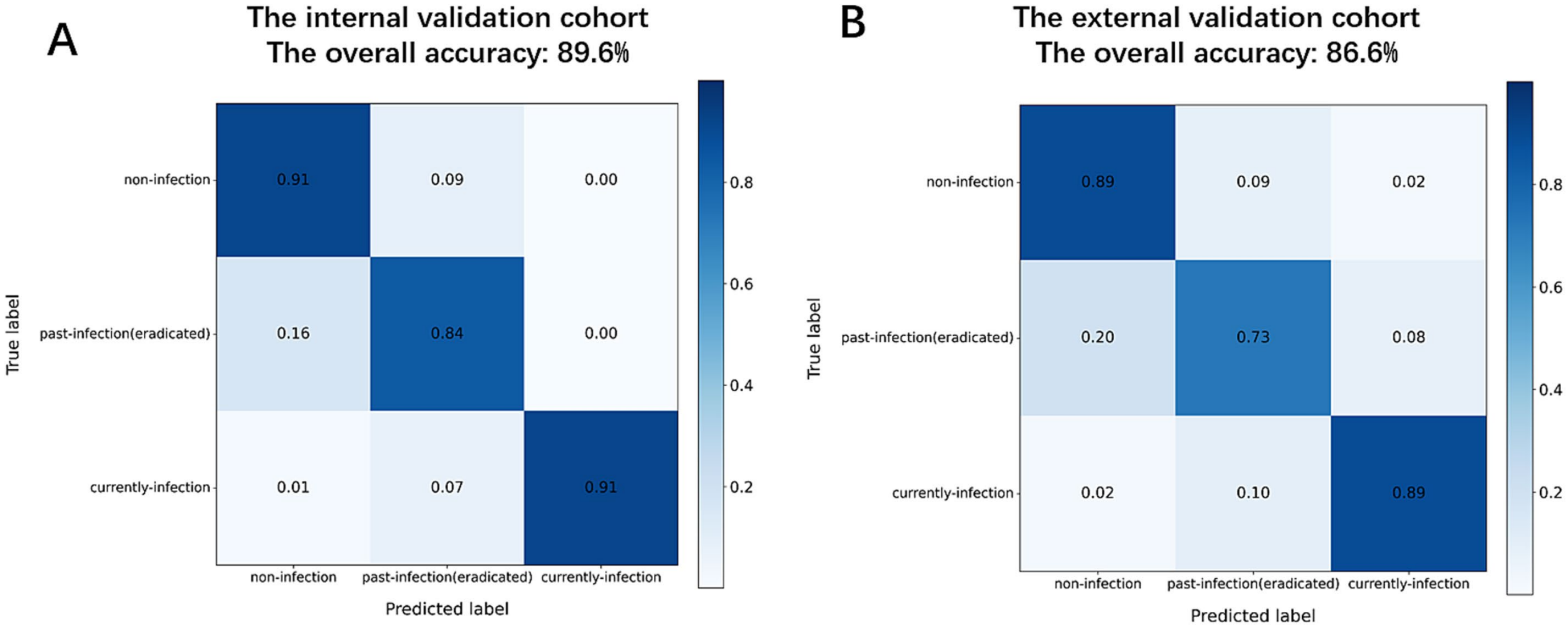
Confusion matrices of diagnostic performance. (**A)** Internal validation cohort. **(B)** External validation cohort.

The MC-CLIP model demonstrated robust and consistent diagnostic performance across both internal and external validation cohorts, (Figure 3), the overall diagnostic accuracy of MC-CLIP was 89.55% (95% CI: 85.5–93.6%) on the internal validation set (197/220) and 85.58% (95% CI: 80.8–90.3%) on the external validation set (178/208). The diagnostic accuracy was 91.4% (internal) versus 89.9% (external) for non-infection, 90.0% vs. 87.0% for past-infection, and 96.4% vs. 94.2% for current-infection. The most significant decline was in past-infection sensitivity (83.7 to 72.5%). Specificity remained stable (91.8% vs. 91.7% for past-infection).

### Comparative analysis of diagnostic performance between MC-CLIP and physicians

#### Internal validation cohort

The MC-CLIP model achieved an overall diagnostic accuracy of 91.4% (95% CI: 87.6–94.2%) for non-infection, 90.0% (85.5–93.4%) for past-infection, and 96.4% (93.1–98.2%) for current-infection, significantly outperforming both senior and junior physicians (Table 2). Notably, MC-CLIP demonstrated superior sensitivity ((83.7% vs. 71.4%; RD: 12.3, 95% CI: 2.1–22.5%; *p* < 0.05)) and PPV (74.5% vs. 59.3 and 44.3%, p < 0.05) for past-Infection and higher specificity across all categories (e.g., 98.7% vs.98.0 and 92.0% for current-infection).

**Table 2:** Comparative diagnostic analysis of MC-CLIP and physicians for *H. pylori’*s three-categorical classification using the internal validation data.

Diagnostic metric	MC-CLIP	Senior physician	Junior physician	MC-CLIP vs. Senior physician	MC-CLIP vs. Junior physician
				RD (95% CI); *P*-value	RD (95% CI); *p*-value
Non-infection					
Sensitivity (95% CI)	91.1% (84.3–95.0)	86.1% (78.3–91.6)	77.2% (68.1–84.4)	5.0% (−2.4, 12.4); 0.187 (n.s.)	13.9% (5.3, 22.5); 0.002 (*)
Specificity (95% CI)	91.6% (85.1–95.4)	89.9% (83.3–94.2)	86.7% (79.3–91.6)	1.7% (−3.8, 7.2); 0.541 (n.s.)	4.9% (0.1 to 9.7); 0.047 (*)
PPV (95% CI)	90.2% (83.1–94.5)	87.9% (80.0–93.0)	83.0% (74.3–89.3)	2.3% (−4.2, 8.8); 0.493 (n.s.)	7.2% (−0.1, 14.5); 0.053 (n.s.)
NPV (95% CI)	92.4% (86.0–96.0)	88.4% (81.5–93.1)	81.7% (74.2–87.5)	4.0% (−1.2, 9.2); 0.132 (n.s.)	10.7% (4.3, 17.1); 0.001 (*)
Accuracy (95% CI)	91.4% (87.6–94.2)	88.2% (83.3–92.0)	82.3% (76.7–86.9)	3.2% (−0.6, 7.0); 0.098 (n.s.)	9.1% (4.4 to 13.8); <0.001 (*)
Past-infection					
Sensitivity (95% CI)	83.7% (71.5–91.3)	71.4% (57.9–82.0)	52.5% (41.5–68.0)	12.3% (0.7 to 23.9); 0.038 (*)	31.2% (18.2 to 44.2); <0.001 (*)
Specificity (95% CI)	91.8% (86.8–95.1)	86.0% (80.0–90.6)	81.3% (73.4–85.6)	5.8% (0.0, 11.6); 0.049 (*)	10.5% (5.2, 15.8); <0.001 (*)
PPV (95% CI)	74.5% (62.3–83.8)	59.3% (46.7–70.7)	44.3% (32.4–56.9)	15.2% (1.7, 28.7); 0.028 (*)	30.2% (17.5, 42.9); <0.001 (*)
NPV (95% CI)	95.2% (90.8–97.5)	91.3% (86.0–94.8)	86.2% (79.9, 90.8)	3.9% (−0.4, 8.2); 0.072 (n.s.)	9.0% (3.6, 14.4); 0.001 (*)
Accuracy (95% CI)	90.0% (85.5–93.4)	82.7% (77.1–87.3)	74.5% (68.4–80.0)	7.3% (1.6, 13.0); 0.012 (*)	15.5% (9.5, 21.5); <0.001 (*)
Current-infection					
Sensitivity (95% CI)	91.4% (82.6–96.0)	84.3% (73.6–91.2)	74.3% (63.2–83.1)	7.1% (−1.7, 15.9); 0.112 (n.s.)	17.1% (8.7, 25.5); <0.001 (*)
Specificity (95% CI)	98.7% (95.2–99.7)	98.0% (94.3–99.3)	92.0% (86.6–95.4)	0.7% (−0.7, 2.1); 0.317 (n.s.)	6.7% (3.4, 10.0); <0.001 (*)
PPV (95% CI)	97.0% (89.5–99.2)	95.2% (86.5–98.5)	81.2% (70.5–88.8)	1.8% (−2.6, 6.2); 0.423 (n.s.)	15.8% (6.2, 25.4); 0.002 (*)
NPV (95% CI)	96.1% (91.8–98.2)	93.0% (87.9–96.2)	88.5% (82.5–92.7)	3.1% (−1.2, 7.4); 0.156 (n.s.)	7.6% (2.6, 12.6); 0.003 (*)
Accuracy (95% CI)	96.4% (93.1–98.2)	93.6% (89.6–96.3)	86.4% (81.2, 90.4)	2.8% (−0.4, 6.0); 0.089 (n.s.)	10.0% (5.4, 14.6) < 0.001 (*)

CI, Confidence Interval; MC-CLIP, Magnetically Controlled Capsule Endoscopy Contrastive Language-Image Pre-Training; NPV, Negative Predictive Value; PPV, Positive Predictive Value; RD, Risk difference; n.s, not significant. Asterisks (*) indicate significant differences (*p* < 0.05).

#### External validation cohort

MC-CLIP maintained robust performance with accuracies of 89.9%.

(85.1–93.4%), 87.0% (81.9–91.0%), and 94.2% (90.1–96.9%) for non-,past-, and current-infection, respectively (Table 3). While the senior physician showed comparable sensitivity for non-infection (91.5% vs. 89.4%, *p* = 0.541), MC-CLIP exhibited significantly and moderately higher sensitivity for current-infection (90.5% vs. 81.0%, *p* = 0.002) and past-infection (72.5% vs. 68.6%, *p* = 0.493) respectively.

**Table 3:** Comparative diagnostic analysis of MC-CLIP and physicians for *H. pylori’*s three-categorical classification using the external validation data.

Diagnostic metric	MC-CLIP	Senior Physician	Junior Physician	MC-CLIP vs. Senior physician	MC-CLIP vs. Junior physician
				RD (95% CI); *P*-value	RD (95% CI); *p-*value
Non-infection					
Sensitivity (95% CI)	89.4% (81.5–94.3)	91.5% (84.4–95.6)	78.7% (69.4–86.1)	−2.1% (−8.9, 4.7); 0.541 (n.s.)	10.7% (2.4, 19.0); 0.012 (*)
Specificity (95% CI)	90.4% (83.6–94.7)	91.2% (84.9–95.1)	83.3% (75.1–89.5)	−0.8% (−6.5, 4.9); 0.783 (n.s.)	7.1%, (0.4, 13.8); 0.038 (*)
PPV (95% CI)	88.4% (80.4–93.6)	89.6% (82.3–94.3)	79.6% (70.5–86.6)	−1.2% (−8.1, 5.7); 0.732 (n.s.)	8.8% (0.1, 17.5); 0.047 (*)
NPV (95% CI)	91.2% (84.6–95.2)	92.9% (86.9–96.3)	82.6% (74.3–88.8)	−1.7% (−6.6, 3.2); 0.493 (n.s.)	8.6% (2.3, 14.9); 0.008 (*)
Accuracy (95% CI)	89.9% (85.1–93.4)	91.3% (86.8–94.5)	81.2% (75.4–86.1)	−1.4% (−6.3, 3.5); 0.572 (n.s.)	8.7% (4.1, 13.3) < 0.001 (*)
Past-infection					
Sensitivity (95% CI)	72.5% (59.1–83.0)	68.6% (55.6–79.5)	54.9% (42.7–66.6)	3.9% (−7.2, 15.0); 0.493 (n.s.)	17.6% (3.1, 32.1); 0.018 (*)
Specificity (95% CI)	91.7% (86.2–95.3)	89.2% (83.3–93.3)	85.4% (78.8–90.3)	2.5% (−4.6, 9.6); 0.493 (n.s.)	6.3% (0.0, 12.6); 0.049 (*)
PPV (95% CI)	74.0% (61.4–83.7)	67.3% (54.8–77.8)	54.9% (42.7–66.6)	6.7% (−5.7, 19.1); 0.287 (n.s.)	19.1% (4.3, 33.9); 0.012 (*)
NPV (95% CI)	91.1% (85.5–94.8)	89.7% (83.9–93.7)	85.4% (78.8–90.3)	1.4% (−3.1, 5.9); 0.541 (n.s.)	5.7% (0.6, 10.8); 0.028 (*)
Accuracy (95% CI)	87.0% (81.9–91.0)	84.1% (78.6–88.6)	77.9% (71.7–83.2)	2.9% (−1.4, 7.2); 0.187 (n.s.)	9.1% (3.4, 14.8); 0.002 (*)
Current-infection					
Sensitivity (95% CI)	90.5% (80.4–96.4)	81.0% (69.6–88.9)	73.0% (61.4–82.3)	9.5% (3.6, 15.4); 0.002 (*)	17.5% (8.9, 26.1); <0.001 (*)
Specificity (95% CI)	95.9% (91.2–98.3)	95.2% (90.3–97.7)	87.6% (81.1–92.2)	0.7% (−2.3, 3.7); 0.655 (n.s.)	8.3% (3.2, 13.4); 0.002 (*)
PPV (95% CI)	90.5% (80.4–96.4)	87.9% (77.5–93.9)	71.9% (60.7–81.0)	2.6% (−5.8, 11.0); 0.541 (n.s.)	18.6% (6.5, 30.7), 0.003 (*)
NPV (95% CI)	95.9% (91.2–98.3)	92.0% (86.5–95.5)	88.2% (81.8–92.7)	3.9% (−0.6, 8.4); 0.089 (n.s.)	7.7% (1.7, 13.7); 0.012 (*)
Accuracy (95% CI)	94.2% (90.1–96.9)	90.9% (86.5–95.5)	83.2% (77.5–87.8)	3.3% (−0.6, 7.2); 0.098 (n.s.)	11.0% (5.7, 16.3); <0.001 (*)

CI, Confidence Interval; MC-CLIP, Magnetically Controlled Capsule Endoscopy Contrastive Language-Image Pre-Training; NPV, Negative Predictive Value; PPV, Positive Predictive Value; RD, Risk difference; n.s, not significant. Asterisks (*) indicate significant differences (*p* < 0.05).

#### Age-stratified subgroup analysis

We performed age-stratified subgroup analysis which further revealed that the diagnostic performance of MC-CLIP for past-infection was significantly lower in elderly patients (≥60 years) compared to younger individuals (<60 years) in both the internal and external validation cohorts (Supplementary Tables 1, 2). Notably, the sensitivity for past-infection declined from 91.9 to 58.3% (*p* < 0.001) in the internal cohort and from 80.0 to 53.9% (*p* = 0.011) in the external cohort among the elderly. In contrast, the model maintained robust and comparable performance for current and non-infection categories across age groups, with no statistically significant decline in sensitivity or specificity observed in most comparisons.

#### Illustrative cases

**Figure 4:**
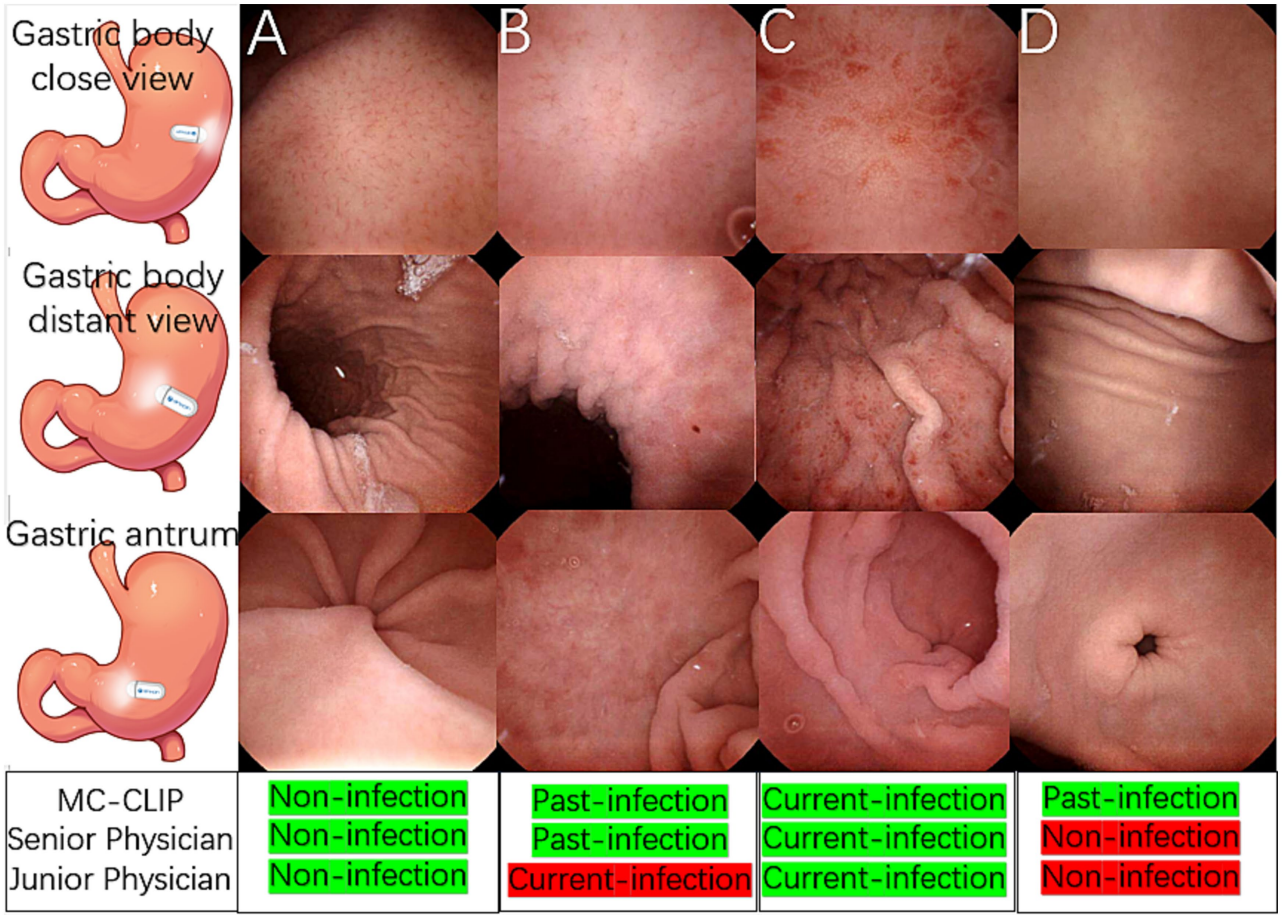
Illustrative cases of *H. pylori* three-categorical classification and a challenging past-infection diagnosis. **(A)** A non-infection case, the gastric body and antrum mucosa was smooth, RAC was clearly observed. **(B)** A past-infection case, map-like redness was observed in gastric body and antrum, the close view of gastric body showed blurred RAC with mild mucosal swelling. **(C)** A current-infection case, mucosal swelling, spotty redness and diffusive redness was observed throughout the gastric body instead of RAC, and erosions was observed in the antrum. **(D)** Distant gastric body image appeared to be normal mucosa, with both physician agreeing on non-infection. Close view of gastric body revealed vague RAC, a diagnostic dilemma between non-infection and past-infection. Antrum appeared normal. MC-CLIP’s collective review of 30 images achieved accurate per-patient diagnosis as past-infection, in contrast to physicians’ erroneous diagnosis of non-infection.

Figures 4A–C presents representative cases of the three distinct *H. pylori* infection statuses, each demonstrating characteristic KCG features. MC-CLIP and the senior physician achieved correct diagnoses for all these 3 cases, but the junior physician misdiagnosed the past-infection case as current-infection.

Figure 4D shows a challenging past-infection case, in which specific finding map-like redness was lacking. MC-CLIP identified subtle mucosal morphological changes and correctly classified this case as past-infection after integrating features across 30 images, whereas both the senior and junior physician, regardless of their level of expertise, misdiagnosed it as non-infection due to isolated ambiguous findings.

## Discussion

AI has been widely used in recent years, and its application in the medical field has had numerous positive impacts, including increasing diagnostic efficiency, promoting health care homogenization, and reducing medical costs (Kual et al., 2020; Ueyama et al., 2021; Seager et al., 2024). Gastrointestinal endoscopy is a key area for the application of AI in the medical field. Commercialized AI systems, including GI Genius (Medtronic), EndoBRAIN (AI Medical Service) and ENDO-AID (Olympus Corporation), that are capable of assisting in the detection of colonic polyps are currently available in clinical practice (Ueyama et al., 2021; Seager et al., 2024; Lau et al., 2024; Iwaya et al., 2023). However, a well-developed and commercialized AI system for the endoscopic three-categorical classification of *H. pylori* infection status has not yet been reported. While existing AI models for endoscopic *H. pylori* detection show promise, two key challenges need to be addressed for improvement.

First, in these studies, the accuracy of the AI’s three-categorical diagnosis of *H. pylori* infection status was significantly lower than that of binary classification. For gastric cancer screening, accurate determination of the past infection status of *H. pylori* via endoscopy is imperative. This type of early-stage gastric cancer can be easily overlooked and requires a comprehensive assessment that includes the morphology of the background mucosa (atrophy and intestinal metaplasia) to assess the risk of gastric cancer (Liu et al., 2024). Furthermore, the endoscopic follow-up strategy for individuals after *H. pylori* eradication is also distinctly different from that for individuals in the other two classification categories. Unfortunately, the diagnostic sensitivity of past infections in previous studies ranged from 40% ~ 65% (Dilaghi et al., 2022,; Jiang et al., 2025), which significantly crippled the performance of gastric cancer screening in real-world practice.

Second, as previously described, traditional CNNs exhibit key.

limitations for *H. pylori* infection diagnosis: (1) fragmented evaluation— analyzing single images in isolation fails to integrate cross-regional features essential for accurate staging, as pathological signs often span multiple gastric zones; and (2) selection subjectivity—dependence on physician image preselection introduces interobserver variability, which is especially challenging for subtle posteradication changes. These constraints hinder reliable three-category classification since clinical diagnosis inherently requires synthesizing findings from diverse anatomical sites, a capability fundamentally lacking in single-image CNN paradigms.

To improve the ability of MCCE to screen for gastric cancer, developing an AI-based three categorical classification of *H. pylori* infection status is necessary but difficult. The quantity of gastric images captured by MCCE far exceeds that of conventional EGD, making the establishment of such an AI classification model much more challenging. Large language models have demonstrated exceptional performance in the realm of AI-assisted pathological diagnosis (Chen et al., 2024a, 2024b). Thus, we are exploring the application of this cutting-edge technology to construct an three categorical *H. pylori* classification model for MCCE.

The MC-CLIP we developed achieved technical breakthroughs in both the training and validation phases. In the training phase, the pretrained model was built on a dataset comprising hundreds of thousands of MCCE cases with greater than 2 million pairs of vision-language alignment, demonstrating significantly improved training efficiency compared with previous CNN models. In the validation phase, MC-CLIP was able to autonomously identify images from a large volume of one individual’s MCCE data according to a previously built *H. pylori* template and output the per-patient result in an end-to-end manner, a diagnostic workflow without the need for physician-led image preselection.

The results of this study showed that our MC-CLIP method achieved highly accurate three-categorical diagnosis of *H. pylori* infection status. The overall accuracies of the internal and external validation cohorts were 89.6 and 85.6%, respectively. The difference was more remarkable regarding the diagnostic performance in patients with past -infection; the sensitivity of MC-CLIP in the internal and external validation cohorts was 83.7 and 72.5%, whereas that of a senior physician was much lower at 71.4 and 68.6%, respectively.

According to the KCG, both current-infection and non-infection patients present with numerous characteristic endoscopic findings, but past-infection patients lack specific endoscopic findings other than map like redness (Xi et al., 2022; Yoshii et al., 2020). Thus, the absence of map-like redness in past-infection individuals poses a significant diagnostic dilemma (Xi et al., 2022; Yoshii et al., 2020). For example, as shown in Figure 4D, the mucosal surface of the gastric body in a previously infected stomach only exhibits subtle changes, whereas the morphology and surface mucosal characteristics of the antrum strongly suggest an uninfected state. Even senior physicians may diagnose this case as non-infection. Unlike the previous CNN models (Shichijo et al., 2017; Nakashima et al., 2020; Seo et al., 2023; Li et al., 2025; Shichijo et al., 2019), which diagnose single images before aggregating results for a per-patient diagnosis, MC-CLIP directly delivers a per-patient diagnosis through joint analysis of 30 selected images, leading to improved diagnostic sensitivity of past infection, which exceeds that of the senior physician in our study and the CNN models in previous studies.

This study has several strengths. First, this multiple-center study is meticulously designed, the sample size well calculated, with an ample volume of data in the training set, and the validation set encompasses both internal and external validation subsets. The diagnostic outcomes of MC-CLIP were compared with those of physicians at different levels of expertise using the internal and external validation set data. Second, this is the first vision–language foundation model (CLIP) for MCCE-based three-categorical *H. pylori* infection classification. Physicians and AI engineers have engaged in deep collaboration, overcoming key limitations of CNN-based approaches and achieving end-to-end automation with 89.6% overall accuracy. Third, our previous work (Xi et al., 2022), in which the applicability of the KCG for MCCE was assessed, laid a solid foundation for the training of the *H. pylori* infection classification model in this study.

This study does have several limitations. First, the moderate decline in diagnostic accuracy observed in the external validation cohort reflects the heterogeneity inherent in real-world clinical practice and underscores the necessity of external validation for assessing model generalizability. Second, subsequent age-stratified analysis revealed significantly reduced sensitivity for diagnosing past-infection among elderly patients (≥60 years). This decline is likely attributable to age-related mucosal changes—such as physiological atrophy, intestinal metaplasia, and medication-induced alterations—which may obscure subtle post-eradication features and complicate accurate classification. Unfortunately, the limited number of past-infection cases in the elderly subgroup precluded more definitive conclusions, highlighting the need for future studies with larger geriatric cohorts to improve model performance in this population.

Furthermore, as the study participants were exclusively recruited from northern urban Chinese populations, the generalizability of MC-CLIP to other ethnicities, geographical regions, and socioeconomic backgrounds remains uncertain and warrants further investigation in diverse demographic settings. Lastly, the spontaneous eradication of *H. pylori* occurs in a certain proportion of the general population, albeit at a very low rate (<1%) (Correa and Houghton, 2007). However, the clinical diagnostic gold standard we employed might have potentially misclassified the true infection status of a few study participants. The potential for misclassification is a limitation of our study and of real-world clinical practice. Future studies with prospective, longitudinal designs and more definitive diagnostic tests could further refine the ground truth.

The MC-CLIP model holds promise for integration into clinical workflows, such as serving as a pre-screening triage tool to prioritize MCCE cases for physician review or as a decision-support system providing real-time annotations during endoscopy interpretation. This could enhance efficiency and reduce the missed diagnosis of subtle morphological changes of past-infection. However, several challenges must be addressed prior to widespread adoption, including the need for regulatory approval, seamless integration with existing hospital information systems, and overcoming the “black-box” nature of deep learning models through explainable AI (XAI) techniques to build clinical trust. Furthermore, ensuring data privacy and security through robust, compliant deployment architectures is paramount.

In the future, we believe that large language models (LLMs) will continue to push the boundaries of AI applications in the medical field (Iwaya et al., 2023; Liu et al., 2024; Leung et al., 2021; Chen et al., 2024a, 2024b). The integration of endoscopic images and multimodal clinical records using LLMs holds promise for the development of superior gastric cancer risk assessment tools, thereby fundamentally reshaping current screening practices.

In conclusion, MC-CLIP demonstrated excellent diagnostic performance, particularly for past-infections, highlighting its strong potential for application in MCCE-based gastric cancer screening.

## Data Availability

The raw data supporting the conclusions of this article will be made available by the authors, without undue reservation.
