# Supplementary material for: AI-powered three-category Helicobacter pylori diagnosis via magnetic controlled capsule endoscopy: a multicenter validation of a vision-language model
Source: Front Microbiol. 2025 Oct 13;16:1687021. doi: 10.3389/fmicb.2025.1687021 (PMC12554734; doi:10.3389/fmicb.2025.1687021)
Supplement: Supplementary file 1 [file Data_Sheet_1.docx]

**Supplemental Table 1. Age-stratified analysis of MC-CLIP’s diagnostic performance of three-categorical classification using the internal validation data**

|  | **Age<60**  (n=175) | **Age≥60**  (n=45) | **RDs(95%CI)** | **P values** |
| --- | --- | --- | --- | --- |
| ***Non-infection*** | | | | |
| **Sensitivity**  **(95% CI)** | 93.8%  (88.5- 99.1) | 81.0 %  (64.2 – 97.8) | 12.8% (0.0, 25.6) | 0.067(n.s.) |
| **Specificity**  **(95% CI)** | 94.8%  (90.3 – 99.2) | 83.3%  (68.4 – 98.2) | 11.4% (-0.4, 23.2) | 0.059(n.s.) |
| **PPV**  **(95% CI)** | 93.8%  (88.5 – 99.1) | 81.0 %  (64.2 – 97.8) | 12.8% (0.0, 25.6) | 0.067(n.s.) |
| **NPV**  **(95% CI)** | 94.8%  (90.3 – 99.2) | 81.0 %  (64.2 – 97.8) | 13.8% (-0.4, 26.6) | 0.059(n.s.) |
| **Accuracy**  **(95% CI)** | 94.3%  (90.9 – 97.7) | 82.2%  (71.0 – 93.4) | **12.1% (3.1, 21.1)** | **0.008(*)** |
| ***Past-infection*** | | | | |
| **Sensitivity**  **(95% CI)** | 91.9%  (83.0 – 96.5) | 58.3%  (35.5 – 78.2) | **33.6% (15.3, 51.8)** | **<0.001(*)** |
| **Specificity**  **(95% CI)** | 93.5%  (88.5 – 96.5) | 84.9%  (71.1 – 92.9) | 8.6% (-1.4,18.7) | 0.093(n.s.) |
| **PPV**  **(95% CI)** | 79.1%  (68.0 – 87.3) | 58.3%  (35.5 – 78.2) | 20.7% (-0.1, 41.6) | 0.052(n.s.) |
| **NPV**  **(95% CI)** | 97.8%  (94.1 – 99.2) | 84.9%  (71.1 – 92.9) | **12.9% (4.4, 21.4)** | **0.003()** |
| **Accuracy**  **(95% CI)** | 93.1%  (88.8 – 96.0) | 77.8%  (65.7 – 86.7) | **15.4% (5.7, 25.0)** | **0.002(*)** |
| ***Current-infection*** | | | | |
| **Sensitivity**  **(95% CI)** | 93.1%  (84.9 – 97.2) | 83.3%  (58.6 – 95.4) | 9.8% (-5.3, 24.8) | 0.204(n.s.) |
| **Specificity**  **(95% CI)** | 99.2%  (95.4 – 99.9) | 96.9%  (84.8 – 99.7) | 2.2% (-1.9, 6.3) | 0.407(n.s.) |
| **PPV**  **(95% CI)** | 98.2%  (91.7 – 99.8) | 90.9%  (65.1 – 98.8) | 7.3% (-5.4, 19.9) | 0.263(n.s.) |
| **NPV**  **(95% CI)** | 96.7%  (92.1 – 98.8) | 94.1%  (80.4 – 98.9) | 2.6% (-4.9, 10.0) | 0.499 (n.s.) |
| **Accuracy**  **(95% CI)** | 97.1%  (93.7 – 98.8) | 93.3%  (83.2 – 97.9) | 3.8% (-3.2, 10.8) | 0.286(n.s.) |

**CI – Confidence Interval, MC-CLIP- Magnetically Controlled Capsule Endoscopy Contrastive Language-Image Pre-Training ,NPV – Negative Predictive Value，PPV – Positive Predictive Value, RD-Risk difference, n.s-not significant**

**Asterisks (*) indicate significant differences (p<0.05).**

**Supplemental Table 2. Age-stratified analysis of MC-CLIP’s diagnostic performance of three-categorical classification using the external validation data**

|  | **Age<60**  (n=164) | **Age≥60**  (n=44) | **RDs(95%CI)** | **P values** |
| --- | --- | --- | --- | --- |
| ***Non-infection*** | | | | |
| **Sensitivity**  **(95% CI)** | 93.2%  (86.5- 96.6) | 75.0 %  (56.3 – 88.2) | **18.2% (5.5, 31.0)** | **0.005(*)** |
| **Specificity**  **(95% CI)** | 92.2%  (86.1 – 96.0) | 83.3%  (65.1 – 93.6) | 8.9% (-3.0, 20.8) | 0.148 (n.s.) |
| **PPV**  **(95% CI)** | 97.8%  (83.3 – 95.3) | 79.0 %  (59.4 – 91.4) | 11.8% (-2.3, 26.0) | 0.099 (n.s.) |
| **NPV**  **(95% CI)** | 94.3%  (88.8 – 97.4) | 80.0 %  (63.2 – 91.1) | **14.3% (3.2, 25.4)** | **0.012 (*)** |
| **Accuracy**  **(95% CI)** | 92.7%  (88.0 – 95.8) | 79.6%  (66.8 – 88.8) | **13.1% (3.6, 22.7)** | **0.008(*)** |
| ***Past-infection*** | | | | |
| **Sensitivity**  **(95% CI)** | 80.0%  (66.6 – 88.0) | 53.9%  (32.8 – 73.7) | **25.1% (5.9, 44.3)** | **0.011(*)** |
| **Specificity**  **(95% CI)** | 93.7%  (88.5 – 96.8) | 83.9%  (68.1 – 93.1) | 9.8% (-1.2, 20.8) | 0.079 (n.s.) |
| **PPV**  **(95% CI)** | 79.0%  (66.6 - 88.0) | 58.3%  (35.5 – 78.2) | **20.6% (1.5, 39.7)** | **0.037(*)** |
| **NPV**  **(95% CI)** | 93.7%  (88.5 – 96.8) | 81.3%  (65.4 – 91.4) | **12.4% (2.3, 22.5)** | **0.017(*)** |
| **Accuracy**  **(95% CI)** | 90.2%  (85.1 – 93.9) | 75.0%  (61.9 – 85.2) | **15.2% (4.1, 26.4)** | **0.008(*)** |
| ***Current-infection*** | | | | |
| **Sensitivity**  **(95% CI)** | 92.3%  (83.5 – 96.8) | 81.8%  (57.1 – 94.5) | 10.5% (-4.6, 25.6) | 0.178 (n.s.) |
| **Specificity**  **(95% CI)** | 98.2%  (94.4 – 99.5) | 87.9%  (73.9 – 95.4) | **10.3% (1.1, 19.6)** | **0.028 (*)** |
| **PPV**  **(95% CI)** | 96.0%  (87.7 – 99.0) | 69.2%  (47.6 – 85.4) | **26.8% (9.7, 43.8)** | **0.002(*)** |
| **NPV**  **(95% CI)** | 96.5%  (92.0 – 98.6) | 93.6%  (80.4 – 98.4) | 2.9% (-5.6,11.5) | 0.497 (n.s.) |
| **Accuracy**  **(95% CI)** | 96.3%  (92.5 – 98.4) | 86.4%  (74.6 – 93.6) | **9.9% (1.3, 18.7)** | **0.025 (*)** |

**CI – Confidence Interval, MC-CLIP- Magnetically Controlled Capsule Endoscopy Contrastive Language-Image Pre-Training ,NPV – Negative Predictive Value，PPV – Positive Predictive Value, RD-Risk difference, n.s-not significant**

**Asterisks (*) indicate significant differences (p<0.05).**
